# WIKI4, a Novel Inhibitor of Tankyrase and Wnt/ß-Catenin Signaling

**DOI:** 10.1371/journal.pone.0050457

**Published:** 2012-12-05

**Authors:** Richard G. James, Kathryn C. Davidson, Katherine A. Bosch, Travis L. Biechele, Nicholas C. Robin, Russell J. Taylor, Michael B. Major, Nathan D. Camp, Kerry Fowler, Timothy J. Martins, Randall T. Moon

**Affiliations:** 1 Department of Pharmacology, Seattle, Washington, United States of America; 2 Institute for Stem Cell and Regenerative Medicine, Seattle, Washington, United States of America; 3 Howard Hughes Medical Institute, Seattle, Washington, United States of America; 4 Quellos High Throughput Screening Core, Seattle, Washington, United States of America; 5 University of Washington School of Medicine, Seattle, Washington, United States of America; 6 Department of Cell and Developmental Biology, University of North Carolina at Chapel Hill, North Carolina, United States of America; 7 KWF Consulting, Seattle, Washington, United States of America; University of Pennsylvania School of Medicine, United States of America

## Abstract

The Wnt/ß-catenin signaling pathway controls important cellular events during development and often contributes to disease when dysregulated. Using high throughput screening we have identified a new small molecule inhibitor of Wnt/ß-catenin signaling, WIKI4. WIKI4 inhibits expression of ß-catenin target genes and cellular responses to Wnt/ß-catenin signaling in cancer cell lines as well as in human embryonic stem cells. Furthermore, we demonstrate that WIKI4 mediates its effects on Wnt/ß-catenin signaling by inhibiting the enzymatic activity of TNKS2, a regulator of AXIN ubiquitylation and degradation. While TNKS has previously been shown to be the target of small molecule inhibitors of Wnt/ß-catenin signaling, WIKI4 is structurally distinct from previously identified TNKS inhibitors.

## Introduction

Wnt family genes encode highly conserved secreted glycoproteins, which activate downstream signal transduction pathways important in development and tissue homeostasis. Wnts can signal through one of several pathways, including the conserved Wnt/ß-catenin pathway. The Wnt/ß-catenin pathway is activated by Wnt ligands binding to Frizzled serpentine receptors and to LRP5/6 co-receptors, leading to the post-translational regulation of the stability of ß-catenin (encoded by *CTNNB1*) (reviewed in [1]). In the absence of a Wnt signal, cytosolic CTNNB1 is bound by the scaffolding proteins Adenomatous Polyposis Coli (APC) and AXIN1, and the kinases Casein Kinase 1 (CSNK1A1) and Glycogen Synthase Kinase (GSK). Sequential phosphorylation of CTNNB1 by CSNK1A1 and GSK3 leads to its recognition by a ubiquitin ligase protein complex and its subsequent degradation by the proteasome. Upon activation of Wnt/ß-catenin signaling, this “destruction complex” is inhibited, resulting in accumulation of newly translated CTNNB1, which then translocates to the nucleus where it acts as a co-activator during transcription of target genes that ultimately lead to context-dependent changes in cell proliferation, specification, or differentiation.

Wnt/ß-catenin-dependent transcription plays critical roles in both embryonic development and in adults [2], [3]. Examination of mice and zebrafish that are transgenic for ß-catenin-dependent reporters has revealed that ß-catenin signaling is spatially and temporally regulated [4]–[9]. Not surprisingly, Wnt/ß-catenin signaling plays many roles in development, including patterning of all three germ layers [10]–[15]. In addition, we and others have shown that ectopic activation of the Wnt/ß-catenin pathway can drive differentiation of human embryonic stem cells (hESCs) towards mesodermal and endodermal lineages [16], [17]. Lastly, Wnt/ß-catenin signaling is activated by acute injury and functions in regenerative responses [18], as well as in diverse chronic diseases including cancers (colorectal cancer [19], liver cancer [20], [21], Wilms tumor [22], [23], lymphoma [24], [25], myeloma [26], [27], [28], leukemias [29], [30]) and neuropsychiatric diseases [31].

There have been a growing number of small molecule inhibitors of Wnt/ß-catenin signaling (reviewed in [32]), which at a minimum should provide tools for modulating the pathway *in vitro*. For example, Huang and colleagues have described a small molecule inhibitor of Wnt/ß-catenin signaling that works by inhibiting the adenosine di-phosphate (ADP) ribosylase protein, Tankyrase (TNKS) [33], [34], [35]. Inhibiting the activity of TNKS leads to elevation of levels of AXIN, thereby promoting the degradation of CTNNB1 and inhibiting Wnt/ß-catenin signaling [33], [34], [35].

In an effort to identify additional small molecule inhibitors of Wnt/ß-catenin signaling, we screened A375 melanoma cells stably transduced with a ß-catenin-activated reporter (BAR). To ensure Wnt pathway-specificity, we cross-screened A375 cells containing luciferase reporters activated by different signaling pathways and eliminated those compounds that inhibited multiple pathways. Using this approach we identified a novel Wnt inhibitor, Wnt Inhibitor Kinase Inhibitor 4 (WIKI4), which effectively blocks Wnt/ß-catenin reporter activity in diverse cell types, including cancer cells that display elevated ß-catenin signaling due to activating APC mutations. WIKI4 inhibits the expression of Wnt target genes as well as the functional effects of Wnt/ß-catenin signaling in colorectal carcinoma cells and hESCs. We subsequently established that WIKI4 antagonizes Wnt/ß-catenin signaling via inhibition of TNKS activity.

## Materials and Methods

### Reagents

The reporters described in this manuscript are lentiviral plasmids containing 12 binding sites for transcription factors downstream of the Wnt/ß-catenin (5′-AGATCAAAGG-3′) (previously described in [36]), Nuclear Factor Kappa B (NF-kB, 5′-GGGAATTTCC-3′), Transforming Growth Factor Beta (TFGß, 5′-AGCCAGACA-3′), and Retinoic Acid (RA, 5′-GGTTCACCGAAAGTTCA-3′) signaling pathways which are each separated by distinct 5-base pair linkers. The transcriptional binding cassettes are located upstream of a minimal thymidine kinase promoter and the firefly open reading frame. Each reporter also contains a separate phosphoglycerate kinase promoter that constitutively drives the expression of a puromycin resistance gene. To engineer stable cell lines that express the reporters, cells were infected with un-concentrated virus, and selected with puromycin (2 µg/mL).

H1 (WiCell) and H1-BAR hESC lines were maintained on irradiated MEF feeders in 20% Knockout Serum Replacement medium +8 ng/ml FGF2 (KSR medium) and passaged weekly using dispase as previously described [17]. NALM6 human pre-B cells (DSMZ) were grown in RPMI 1640 with 10% fetal bovine serum (FBS) and 55 µM ß-mercaptoethanol, A375 malignant melanoma cells (ATCC) were grown in RPMI 1640 with 5% FBS. DLD1 colorectal carcinoma (ATCC), SW480 colorectal carcinoma (ATCC), U2OS osteosarcoma (ATCC) cells were grown in DMEM/F12 with 10% FBS. Wnt3A conditioned medium (CM) and control L CM were generated as previously described [17] from L cells and L-Wnt3A cells (ATCC).

The following antibodies were used: AXIN1 (2087; Cell Signaling Technology), AXIN2 (2151; Cell Signaling Technology), CTNNB1 (9562; Cell Signaling Technology), S33/37T41P-CTNNB1 (9561; Cell Signaling Technology), S45P-CTNNB1 (9564; Cell Signaling Technology), GCTM2 (kind gift from Martin Pera, University of Melbourne, Australia; GCTM-2 antibody previously described in [37], [38], [39]), CD9 (mAB4427; Millipore), TUBB1 (T7816; Sigma-Aldrich), UBIQUITIN (SC-8017, Santa Cruz Biotechnology). The following compounds were used in this study: MG132 (474790, EMD Millipore), XAV-939 (S1180, Selleck Chemical), U0126 (U-6770, LC Labs), and WIKI4 (7990417, Chembridge). All sequences used for real-time PCR or siRNA transfection are listed in Table S1 or previously published [17]. All molecules used in the structure activity relationship analysis are detailed in Table S2.

### High Throughput Small Molecule Screen

Screening was performed using the facilities of the Quellos High Throughput Screening Facility at the Institute for Stem Cell and Regenerative Medicine in Seattle, WA. Compounds dissolved in DMSO were obtained from Chembridge (a custom selection of 6,492 entities from Chembridge’s KINASet library). For the primary screen, performed in duplicate, A375 malignant melanoma cells stably expressing BAR were cultured in growth medium (DMEM/5%FBS/1%antibiotic). 4000 cells per well were transferred to clear bottom 384-well plates (BD Falcon; Fisher Scientific 08-772-004) in 30 µL of growth media using a Matrix WellMate (ThermoScientific). The following day 50 nL of each compound (final concentrations of 370 nM and 10 µM) and 10 µL of Wnt3A-conditioned media (EC_20_ dose) was transferred to the cells. On the third day, 10 µL of resazurin (final concentration 0.1 mg/mL) was added to the cells, and after a three hour incubation viability was assessed by quantifying the fluorescent reduction product of resazurin using an Envision Multilabel plate reader (PerkinElmer). Finally, 5 µL of Steady-Glo (Promega) was added to each well, and luciferase was quantified using the Envision Multilabel plate reader. The fold-increase over the background of DMSO controls for viability and luciferase was calculated. Inhibitors were chosen for further analysis if they inhibited Wnt3A-dependent luciferase production at the 370 nM concentration (normalized Steady-Glo fold change <0.5) but did not decrease resazurin reduction at the 10 µM concentration (viability fold change >0.9). For the Z-factor calculation (Z′), we used the following equation [40]:
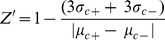



In this equation µ equals the mean calculated from the positive (c+) and negative (c-) control replicates, and σ equals the standard deviation calculated from the positive (c+) and negative control replicates (c-). For the secondary screening, compounds that satisfied our hit criteria were re-screened for their ability to inhibit stimulation of reporters for the Wnt/ß-catenin, NF-kB, TGFß, and RA pathways stably expressed in A375 melanoma cells as described above.

### Functional Cell Assays

For the DLD1 colony forming assays, single DLD1 cells were plated at 1000 cells per well (6-well) and cultured overnight in DMEM containing 0.5% FBS. The next day, compounds were added to the media, and the cells were subsequently cultured for ten days with refreshment of the media and compound occurring every two days. At the end of the culture period, the colonies in each well were counted.

For the flow cytometric analysis of cell surface markers in hESCs, H1-BAR-VENUS hESCs were seeded as small clusters on MEFs in KSR media at ∼35,000 cells per square cm. The following day, the medium was replaced with 50% (vol/vol) conditioned medium (control L or Wnt3A CM) in KSR media with or without inhibitors, XAV-939 or WIKI4. DMSO served as a vehicle control for the compounds. The medium was replenished daily. After 6 days of treatment, hESCs were isolated as single cells with TrypLE Express (Invitrogen) and counted. 500,000 cells were immunolabeled with 100 ul primary antibodies: GCTM2 (hybridoma supernatant, 1∶2, a kind gift from Martin Pera, University of Melbourne, Australia; GCTM-2 antibody previously described in [37], [38], [39]) and CD9 (TG30 clone, 1∶100, Millipore). Cells were then incubated with isotype-specific secondary antibodies (Invitrogen): goat anti-mouse IgM-Alexa 647 (1∶100) and goat anti-mouse IgG2a-biotin (1∶5000), followed by PE-Cy7-streptavidin (1∶250). hESCs were resuspended in 140 ng/ml DAPI in KSR media, then passed through a cell strainer prior to analysis on a BD FACSCanto II flow cytometer. Results were quantified using FlowJo software. The percentage of GCTM2 and CD9 double-positive hESCs was determined from the DAPI-negative (viable), DsRED-positive gated population. DsRED is constitutively expressed in H1-BAR-VENUS cells, thus this gating strategy serves to exclude any MEFs.

For the gene expression analysis in hESCs, total RNA was isolated using TRIZOL according to the manufacturer’s protocol (Invitrogen). 2.5 µg RNA was used for cDNA synthesis with RervertAid First Strand cDNA Synthesis kit (Fermentas). cDNA was diluted 100-fold, then used as template for quantitative PCR (2 µl cDNA per 10 µl reaction) using Applied Biosystems SYBR Green-based detection according to the manufacturer’s protocol on a Roche Lightcycler 480 instrument. Duplicate reactions were performed for each sample. Transcript copy numbers were normalized to GAPDH for each sample, and fold expression over the untreated control was calculated for each gene of interest. Primer sequences are previously published [17] or listed in Table S1.

For all low-throughput siRNA experiments, siRNAs were reverse-transfected at a final concentration of 10 nM using RNAiMAX (#13778-075, Invitrogen) according to the manufacturer’s instructions.

### Biochemistry

For the compound wash-off experiments, ten million SW480 or DLD1 colorectal carcinoma cells were treated overnight with DMSO (D9170; Sigma-Aldrich), WIKI4, or XAV-939 at the concentrations indicated. Cells were then washed off and treated for one hour with the indicated treatments, and then lysed in RIPA buffer (50 mM Tris-cl pH 7.4, 150 mM NaCl, 1% NP40, 0.25% Na-deoxycholate, 5 mM ADP-HPD and 5 mM N-ethyl maleimide). Lysates were immunoprecipitated overnight with the indicated antibodies and analyzed by western blot. The *in vitro* TNKS2 assay was acquired from commercial sources (80565; BPS Bioscience).

## Results and Discussion

### Identification of WIKI as a Small Molecule Inhibitor of Wnt/ß-catenin Signaling

**Figure 1:**
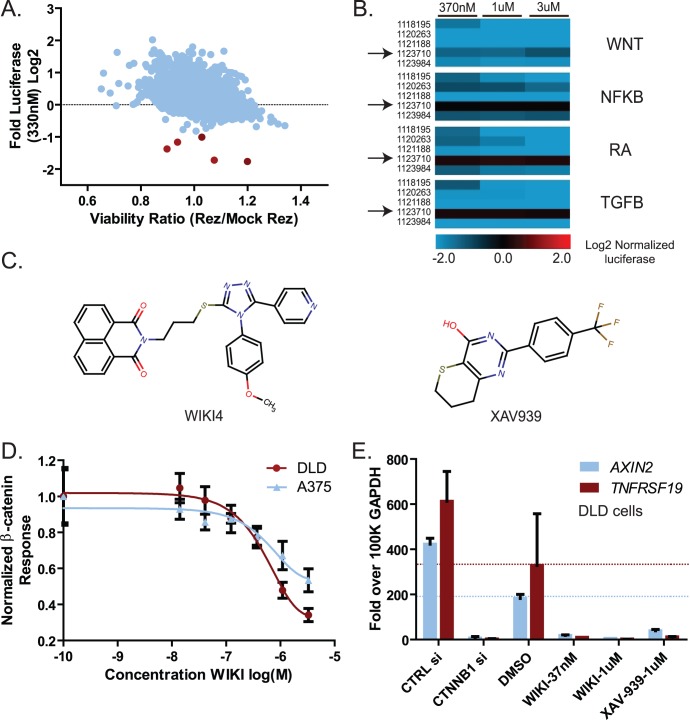
WIKI4 is identified as a novel small molecule inhibitor of the Wnt/ß-catenin pathway. (**A**) Scatter plot of a small molecule screen in human A375 melanoma cells stably expressing the ß-catenin Activated Reporter (BAR) driving firefly luciferase with each dot representing a single compound. The red dots represent compounds that exhibited decreased luciferase signal (> two standard deviations below the sample mean), and unchanged cell viability as measured by resazurin. (**B**) A heat map showing the effects of five Wnt/ß-catenin inhibitors on reporters for the Wnt/ß-catenin, Nuclear Factor Kappa B (NF-kB), Retinoic Acid (RA), and Transforming Growth Factor ß (TGFB) pathways. WIKI4 (arrow) is the only compound that specifically inhibits Wnt/ß-catenin signaling. (**C**) Chemical structure of WIKI4 (left) and XAV-939 (right). (**D**) Dose response curves showing that WIKI4 inhibits ß-catenin reporter activity in DLD1 colorectal carcinoma cells and Wnt-stimulated A375 melanoma cells. (**E**) Inhibition of the expression of the ß-catenin target genes *AXIN2* and *TNFRSF19* by WIKI4 as assessed by quantitative PCR. DLD cells were transfected with CTNNB1 siRNA as a control 72 hours prior to harvesting for RNA; cells were treated with compounds or DMSO for 16 hours prior to harvesting. The experiments in (**D**) and (**E**) are representative of three independent experiments and the error bars represent standard deviation from four technical replicates.

To make an assay for Wnt/ß-catenin signaling suitable for high throughput screening, we generated A375 melanoma cells stably infected with a ß-catenin-activated luciferase reporter (BAR) [23], [36] and selected populations in which luciferase activity is increased at least 4,000-fold by WNT3A. We tested the robustness of our assay by calculating the Z-factor (Z′) values [40] using probes that are known to enhance (U0126 [41], Riluzole [42], and GSK3B inhibitor IX [43]) or inhibit (XAV-939 [33]) Wnt/ß-catenin signaling (Figure S1A). For all control probes, we found the Z′ values to be greater than .45 (Figure S1A), a value considered robust in high throughput screening assays [40]. Following validation of our assay, we then screened A375 melanoma cells at two concentrations of a small molecule library in the presence of a twenty percent effective concentration (EC_20_) dose of WNT3A. We focused on small molecules that reduced expression of the luciferase reporter at a low dose (330 nM) and that did not kill cells at a high dose (10 µM) relative to controls treated with dimethyl sulfoxide (DMSO), with the expectation that these criteria would filter out compounds that inhibited BAR due to cellular toxicity. Five compounds met our criteria for further study by significantly decreasing Wnt/ß-catenin signaling without causing toxicity at either dose (Fig. 1A).

We next asked whether any of the five compounds preferentially modulated Wnt/ß-catenin signaling by comparing the repression of BAR in A375 cells relative to luciferase reporters for the Nuclear Factor Kappa B (NF-kB), Transforming Growth Factor Beta (TGFß), and Retinoic Acid (RA) signaling pathways (Fig. 1B). Of the five candidate Wnt/ß-catenin inhibitors that we tested, WIKI4 (left panel, Fig. 1C) was the only inhibitor of BAR that did not also inhibit the reporters for NF-kB, TGFß, and RA (Fig. 1B). Furthermore, WIKI4 has demonstrated activity in one of nine published assays (http://pubchem.ncbi.nlm.nih.gov/summary/summary.cgi?cid=2984337), supporting our contention that WIKI4 is not a general inhibitor of activity in high throughput screening assays. We then demonstrated that WIKI4 inhibits Wnt/ß-catenin signaling in several other cell lines, including DLD1 colorectal cancer cells (Fig. 1D), NALM6 B cells (Figure S1B), U2OS osteosarcoma cells (Figure S1B) and hESCs (Figure S1C). In all cell types tested, we observed that WIKI4 potently inhibited Wnt/ß-catenin signaling and that its half-maximal response dose was ∼75 nM.

We next investigated whether WIKI4 is sufficient to inhibit expression of Wnt/ß-catenin target genes in DLD1 colorectal carcinoma cells, which express a truncated form of the Wnt/ß-catenin inhibitor APC [44]. We found that incubation of DLD1 cells overnight with either WIKI4 or the structurally distinct TNKS inhibitor, XAV-939 (right panel, Fig. 1C) [33], resulted in decreased steady-state abundance of *AXIN2,* and *TNFRSF19* (Fig. 1E), which is consistent with WIKI4 acting as an inhibitor of Wnt/ß-catenin signaling. Furthermore, we observed that WIKI4 is sufficient to inhibit WNT3A-dependent increases in the expression of *AXIN2* and *TNFRSF19* in hESCs (Figure S1D, S1E). Thus we have identified WIKI4 as a new inhibitor of Wnt/ß-catenin signaling that regulates the pathway in several cell types.

To determine which chemical groups in WIKI4 are required for its ability to inhibit Wnt/ß-catenin signaling, we next performed a structure activity relationship analysis (Figure S2). WIKI4 has a molecular weight of 522 and a calculated partition coefficient of 4.8, putting it near the limits of “druglikeness” by Lipinski’s Rule of Five [45]. WIKI4’s mass and complexity is greater than XAV-939 (Fig. 1C), and identification of small active WIKI4 analogs could provide more opportunities for modification while maintaining its druglike properties. To identify less complex WIKI4 analogs and to determine which portions of WIKI4 are required for activity, we searched for commercially available analogs. We queried the ZINC [46] and eMolecule (www.emolecules.com) databases and identified 62 WIKI4 analogs for further testing (Table S3). We assayed the Wnt/ß-catenin inhibitory activity of a subset of these compounds (Figure S2). Our results indicate that the traizole’s 4-pyridyl and 4-methoxyphenyl groups tolerate some modification, but the latter group could not be removed (Figure S2A). Additionally, substitution of the 1,8-naphthalimide group with a phthalimade group eliminated activity as did replacement of the 1,8-naphthalimide group with a methyl or phenyl group (Figure S2B).

### WIKI4 Inhibits the Cellular Responses to Wnt/ß-catenin Signaling

**Figure 2:**
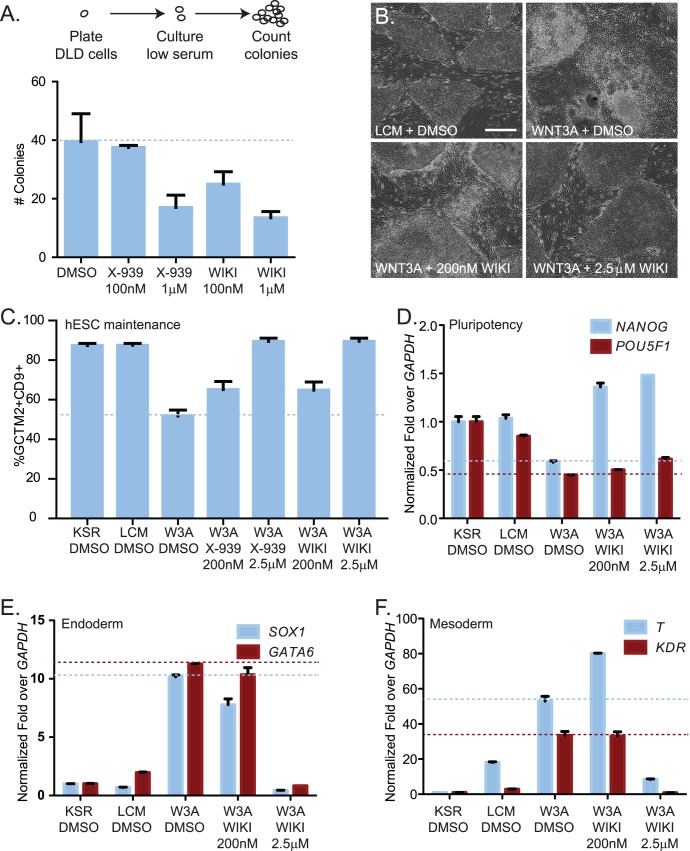
WIKI4 inhibits the functional outcomes of Wnt/ß-catenin signaling. (**A**) WIKI4 inhibits colony formation of DLD1 colorectal cancer cells. DLD1 cells were plated individually in 0.5% serum containing medium, and treated with the indicated concentrations of WIKI4 and XAV-939. This experiment is representative of three independent experiments and the error bars represent standard deviation of three technical replicates. (**B-F**) WIKI4 prevents Wnt3A-dependent differentiation of H1 human embryonic stem cells (hESCs). (**B**) Culturing hESCs for six days with Wnt3A causes marked morphological changes that are rescued by treatment with WIKI4. Scalebar  = 500 µm. (**C**) Treatment with WIKI4 prevents the decrease in co-expression of markers of undifferentiated hESCs following Wnt3A stimulus. hESCs were stimulated with the indicated treatments and expression of GCTM2 and CD9 was assessed by flow cytometry following six days of treatment. (**D-F**) The effect of WIKI4 treatment on the expression of genes that are altered during Wnt3A-dependent differentiation of hESCs was assessed by qPCR. hESCs were treated for the indicated conditions for six days, and then analyzed by qPCR for markers of undifferentiated stem cells (*NANOG, POU5F1*) (**D**), endoderm (*SOX17, GATA6*) (**E**), and mesoderm (*T, KDR*) (**F**). The data was normalized to 100,000 copies of *GAPDH* and plotted as a ratio to the untreated hESCs (cultured in KSR media). The data in the experiments presented in **B-F** are representative of three independent experiments and the error represents standard deviation of technical replicates. In **B-F**, LCM = control L cell CM, WNT3A = Wnt3a CM; both 50% (vol/vol) in KSR medium.

We next asked whether cells treated with an effective dose of WIKI4 would show a reduction in Wnt/ß-catenin-mediated responses at the cellular level. As DLD1 colorectal cancer cells require ß-catenin signaling for growth in limiting culture experiments [47], these cells provide an excellent functional model of the pathway in which to test small molecules. We found that WIKI4 inhibits growth of DLD1 cells relative to DMSO controls in media containing low serum (Fig. 2A). This result demonstrates that WIKI4 inhibits a known cellular response to Wnt/ß-catenin signaling.

Given that cellular responses to Wnt/ß-catenin signaling are diverse and context-dependent, we next examined the effects of WIKI4 on hESCs. Activation of Wnt/ß-catenin signaling in hESCs alters their cell fate and causes them to differentiate into early mesoderm and endoderm lineage cells [16], [17]. Upon stimulation with Wnt3A for 6 days, hESC colonies exhibit overt phenotypic changes that include loss of compact colony structure (top panels, Fig. 2B), decreased co-expression of cell surface markers of undifferentiated hESCs (GCTM2 and CD9, Fig. 2C) and decreased steady-state RNA abundance of pluripotency genes (*NANOG* and *POU5F1*, Fig. 2D). Additionally, treatment of hESCs with WNT3A leads to increased expression of genes associated with endoderm (*SOX17* and *GATA6,* Fig. 2E) and mesoderm (*T* and *KDR,* Fig. 2F) differentiation. We found that in hESCs treated with both Wnt3A and WIKI4, the WNT3A-dependent effects that we typically observe on colony morphology (bottom panels, Fig. 2B), expression of cell surface markers (Fig. 2C) and expression of markers of pluripotency and differentiation (Fig. 2D, 2E, 2F) were eliminated. We conclude that WIKI4 inhibits Wnt/ß-catenin-mediated processes in hESCs, as well as in DLD1 cells, suggesting that WIKI4 acts on a conserved component of the Wnt/ß-catenin signaling pathway.

**Figure 3:**
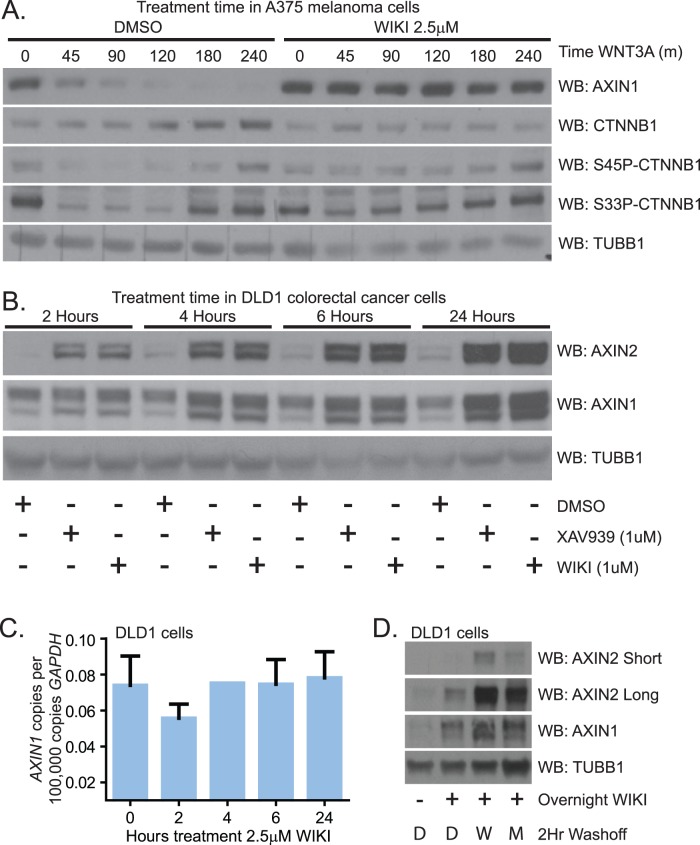
WIKI4 increases the steady-state abundance of the Wnt/ß-catenin inhibitory protein, AXIN1. (**A**) WIKI4 prevents degradation of AXIN1 following stimulation with Wnt3A. A375 melanoma cells were stimulated with 10% (vol/vol) Wnt3A CM for the indicated time periods with or without WIKI4 treatment, lysed and analyzed by western blot using the indicated antibodies. (**B**) WIKI4 increases the steady-state abundance of AXIN1 and AXIN2 protein. DLD1 colorectal carcinoma cells were incubated with DMSO, WIKI4 or XAV-939 for the indicated times, lysed and analyzed by western blot. (**C**) WIKI4 does not significantly affect the steady-state RNA abundance of *AXIN1.* DLD1 colorectal carcinoma cells were incubated with WIK4 for the indicated times, and processed for qPCR to assess changes in the steady-state abundance of *AXIN1* transcript. This data is representative of two independent experiments and the error bars represent standard deviation. (**D**) WIKI4-dependent increases in AXIN1 protein abundance can be maintained by treatment with a proteasome inhibitor. DLD1 colorectal carcinoma cells were treated overnight with WIKI4, and after washing were then incubated for two hours with DMSO (D), WIKI4 (W), or the proteasome inhibitor MG132 (M). The cells were lysed and analyzed by western blotting for the indicated antibodies.

### WIKI4 Increases Steady-state Abundance of AXIN1

After stimulation of A375 melanoma cells with Wnt3A, we observed that the steady-state abundance of the scaffold protein AXIN1 is reduced (left time course, Fig. 3A) and conversely, abundance of cytosolic CTNNB1 increases (left time course, Fig. 3A). Additionally, we observed that the abundance of CTNNB1 that is phosphorylated at sites that are regulated by the destruction complex components CSNK1A1 (S45, left time course Fig. 3A) and GSK3B (S33, left time course, Fig. 3A) is decreased following Wnt3A stimulation. We next investigated whether WIKI4 regulates the biochemical changes associated with Wnt/ß-catenin signaling. We found that WIKI4 inhibits WNT3A-dependent increases in the steady-state abundance of cytosolic CTNNB1, inhibits Wnt3A-dependent decreases in steady-state abundance of AXIN1, and inhibits Wnt3A-dependent decreases in abundance of phosphorylated of ß-catenin (S33 and S45) (right time course, Fig. 3A). Taken together, our findings indicate that WIKI4 modulates Wnt-dependent changes in the abundance and phosphorylation of known core components of the Wnt/ß-catenin signaling pathway.

We next examined whether WIKI4 alters steady-state abundance of AXIN1 and the related AXIN2 in another cell type. Increases in the steady-state abundance of the AXIN scaffolding proteins have been shown to correlate with decreases in the steady-state abundance of cytosolic CTNNB1, even in APC-mutant colon cancer cells [33], [34]. To test the effects of WIKI4 on AXIN levels in APC-mutant cells, DLD1 colorectal cancer cells were treated with WIKI4 for two, four, six or 24 hours and processed for western blotting. We observed that WIKI4 significantly increased the steady-state abundance of AXIN1 and AXIN2 (Fig. 3B) to levels similar to those seen with treatment with the TNKS inhibitor XAV-939.

To further investigate how WIKI4 regulates AXIN protein abundance, we queried whether WIKI4 treatment promotes expression of *AXIN* mRNA or whether it prevents the degradation of AXIN by the proteasome. Using quantitative PCR (qPCR) analyses of DLD1 colorectal carcinoma cells, we found that steady state levels of *AXIN1* (Fig. 3C) and *AXIN2* (Fig. 1D) transcripts were not increased upon treatment with WIKI4. To test whether WIKI4 inhibits AXIN protein turnover, we treated DLD1 cells overnight with WIKI4, and then released them from treatment the next day for two hours (wash-off). We found that cells continuously treated with WIKI4 during the wash-off period exhibited increased abundance of AXIN1 and AXIN2 relative to cells treated with DMSO (Fig. 3D, compare lanes two and three), suggesting that WIKI4 prevents turnover of the AXIN proteins. When DLD1 cells were treated with the proteasome inhibitor MG132 during the wash-off period, AXIN1 and AXIN2 protein abundance remained elevated (Fig. 3D, compare lanes 3 and 4). Taken together, the qPCR and wash-off experiments suggest that WIKI4 increases the steady-state abundance of AXIN proteins by preventing their degradation by the proteasome.

### WIKI4 Blocks the Activity of TNKS2 and Prevents AXIN Ubiquitylation

**Figure 4:**
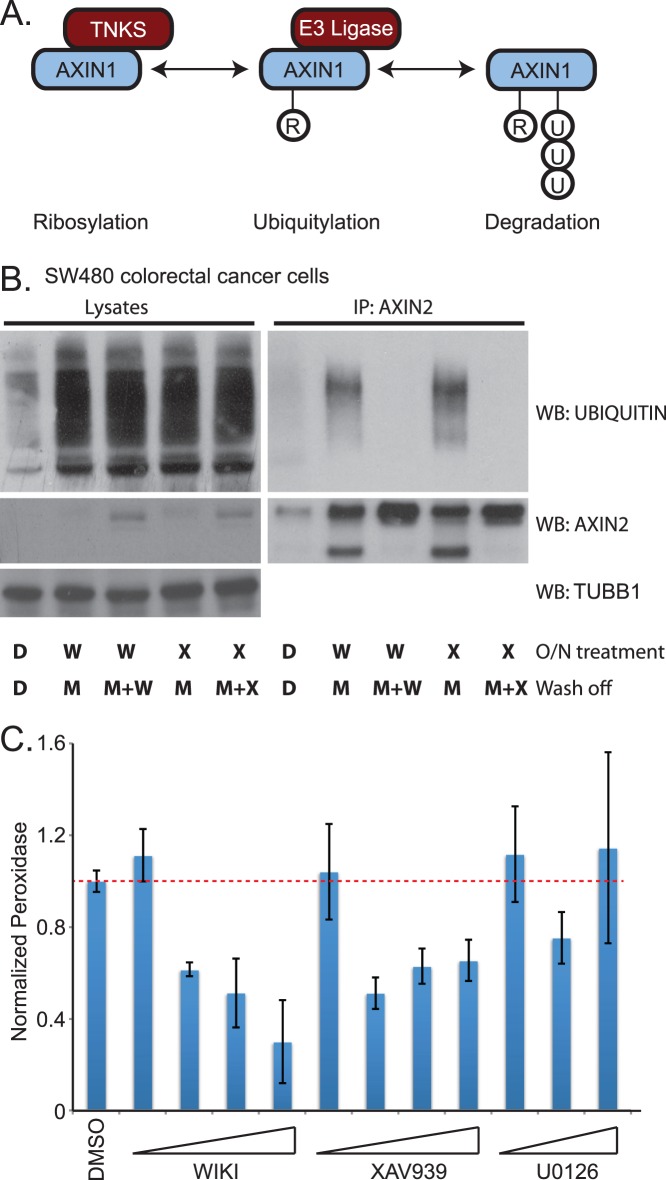
WIKI4 prevents ubiquitylation of AXIN and inhibits the enzymatic activity of TNKS2. (**A**) Schematic showing a model of how AXIN proteins are sequentially ADP-Ribosylated and then poly-ubiquitylated prior to their degradation by the proteasome. (**B**) WIKI4 inhibits ubiquitylation of AXIN2 in SW480 colorectal carcinoma cells. SW480 cells were treated overnight with DMSO (D), 2.5 µM WIKI4 (W) or 2.5 µM XAV-939 (X). Following a brief wash, the cells were then incubated for two hours with DMSO (D), 10 µM MG132 (M) or MG132 and one of the Wnt/ß-catenin pathway inhibitors. Lysates and AXIN2 immunoprecipitates from this experiment were processed for western blotting with the indicated antibodies. (**C**) WIKI4 inhibits the enzymatic activity of TNKS2. Recombinant GST-TNKS2 was bound to 96-well plates coated with glutathione. Auto-ADP-ribosylation assays were carried out using biotinylated substrate in the context of the indicated treatments. The amount of TNKS2 auto-ribosylation was quantified by performing chemiluminescent detection of the reaction between streptavidin conjugated to horseradish peroxidase and biotinylated substrate. U0126 was used as a negative control.

AXIN1 is modified sequentially by two enzymes in order for it to be recognized by the proteasome for degradation. First, AXIN1 is ADP-ribosylated by the TNKS1 and TNKS2 enzymes [33]. Subsequently, ADP-ribosylated AXIN1 is bound by the E3 ubiquitin ligase RNF146, which specifically catalyzes its ubiquitylation (Fig. 4A, [48], [49]). To test whether WIKI4 prevents ubiquitylation of AXIN protein, we treated SW480 (Fig. 4B) and DLD1 (Figure S3A, S3B) colorectal carcinoma cells overnight with WIKI4, and subsequently incubated for two hours with either MG132 alone or MG132 and WIKI4 (wash-off). We found that inhibition of the proteasome during the wash-off period with MG132 led to an increase in the abundance proteins bound to ubiquitin (Fig. 4B, Fig. S3A, S3B left panels). We further observed that WIKI4 treatment during the wash-off period reduced the detection of ubiquitin in immunoprecipitated AXIN2 (Fig. 4B, Figure S3A) and AXIN1 (Figure S3B), suggesting that WIKI4 indeed inhibits AXIN ubiquitylation.

One possible explanation for WIKI4-dependent inhibition of AXIN ubiquitylation is that WIKI4 directly inhibits TNKS-mediated ADP-ribosylation of AXIN. The ADP-ribosylation activity of TNKS proteins can be assayed *in vitro* by quantifying their ability to catalyze auto ADP-ribosylation. To investigate the hypothesis that WIKI4 inhibits the catalytic activity of TNKS proteins, we performed *in vitro* auto-ADP-ribosylation assays using recombinant TNKS2. Similar to what is observed for the known TNKS inhibitor XAV-939, we found that WIKI4 prevents auto-ADP-ribosylation of TNKS2 at an IC50 of ∼15 nM (Fig. 4C). In contrast to the effects of XAV-939 and WIKI4, a second ATP analog, U0126, failed to inhibit auto-ADP-ribosylation of TNKS2, demonstrating that our assay is specific (Figure 4C). Taken together, our data suggest that WIKI4 inhibits Wnt/ß-catenin signaling by inhibiting tankyrase activity, and thus preventing the ubiquitylation and degradation of AXIN proteins.

### Conclusions

In summary, we have identified and characterized WIKI4, a novel small molecule inhibitor of Tankyrase that leads to inhibition of Wnt/ß-catenin signaling in multiple cell lines and in hESCs. As the structure of WIKI4 is distinct from the other published Tankyrase inhibitors [33], [34], [35], it is unlikely to share off-target effects with those molecules. Therefore, WIKI4 will be useful as a complementary biological probe for researchers who wish to inhibit the Wnt/ß-catenin pathway by inhibiting Tankyrase.

## Supporting Information

Figure S1 **WIKI4 inhibits Wnt/ß-catenin signaling in several cell types.** (**A**) A375 melanoma cells stably expressing the ß-catenin activated reporter were stimulated with an EC_20_ dose of Wnt3A and treated with the indicated doses of U0126, Riluzole, GSK3 inhibitor IX (left), and XAV-939 (right). The fold change in reporter activity was plotted and the calculated Z-factor (z′) for each compound is indicated above the bars. (**B**) A heat map showing the inhibitory effects of WIKI4 on Wnt3A-dependent activation of the ß-catenin activated reporter stably transduced in NALM6 B cells, U2OS osteosarcoma cells, and A375 melanoma cells. The results of the firefly luciferase and resazurin activity were normalized to the Wnt3A-stimulated condition, log2 transformed and plotted. (**C**) H1 hESCs were stably transduced with the ß-catenin activated reporter (BAR) or a mutated ß-catenin activated reporter (FUBAR) driving firefly luciferase. Both cell lines were stimulated with 50% (vol/vol) Wnt3A CM for three days in the presence of a dose curve of WIKI4. The cells were lysed and the ratio of luciferase from the BAR and FUBAR cells was calculated and plotted. (**D, E**) Wnt3A-dependent increases in the steady-state abundance of gene targets of the Wnt/ß-catenin pathway were prevented by concurrent treatment with WIKI4. hESCS were stimulated for three days with the indicated conditions. The cells were lysed and processed for qPCR for *AXIN2* (**D**) and *TNFRSF19* (**E**). The data was normalized to 100,000 copies of *GAPDH* and plotted as a ratio to the treatment cultured in KSR media.
(PDF)

Figure S2 **Structure activity relationship of WIKI4 analogs.** The Wnt/ß-catenin inhibitory activity of several WIKI4 analogs was tested. The portion of the molecule that is held constant throughout the analysis is depicted in the left panels and the structure specific to each indicated analog is depicted in the right panels. DLD1 colorectal carcinoma cells stably expressing the ß-catenin Activated Reporter (BAR) were treated with a dose escalation of the indicated WIKI analogs. If the compound inhibited signaling, the full dose response curve is depicted, if the compound exhibited no activity, “no response” was indicated, and if the data we have for the compound came from the primary screen, its activity at 330 nM was indicated. (**A**) Modification of the triazole of WiKI4. (**B**) Modification of the 1,8-naphthalimide of WIKI4.
(PDF)

Figure S3 **WIKI4 inhibits polyubiquitylation of AXIN proteins in DLD1 colorectal carcinoma cells.** WIKI4 inhibits ubiquitylation of AXIN2 (**A**) and AXIN1 (**B**) in DLD1 colorectal carcinoma cells. DLD1 cells were treated overnight with DMSO (D), 2.5 µM WIKI4 (W) or 2.5 µM XAV-939 (X). Following a brief wash, the cells were then incubated for two hours with DMSO (D), 10 µM MG132 (M) or MG132 and one of the Wnt/ß-catenin pathway inhibitors. Lysates and AXIN2 (**A**) or AXIN1 (**B**) immunoprecipitates from this experiment were processed for western blotting with the indicated antibodies.
(PDF)

Table S1 **Quantitative PCR primers and siRNA sequences.**
(DOCX)

Table S2 **Compounds tested for structure activity relationship.**
(DOCX)

Table S3 **WIKI analogs identified from public databases.**
(DOCX)
