# Supplementary figures and images for: WIKI4, a Novel Inhibitor of Tankyrase and Wnt/ß-Catenin Signaling
Source: PLoS One. 2012 Dec 5;7(12):e50457. doi: 10.1371/journal.pone.0050457 (PMC3515623; doi:10.1371/journal.pone.0050457)

A.

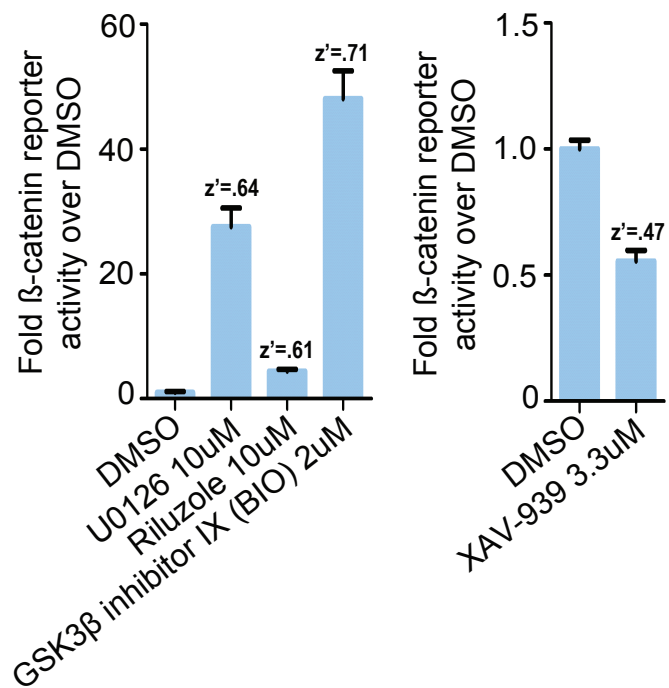

B.

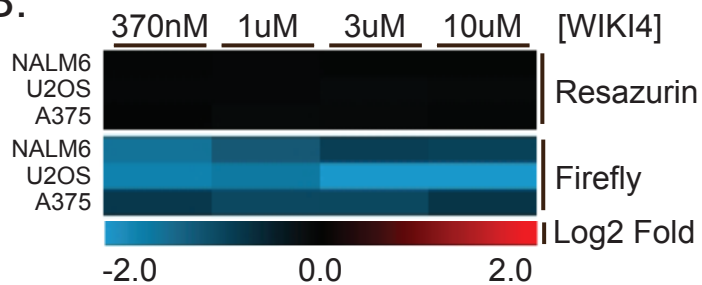

C.

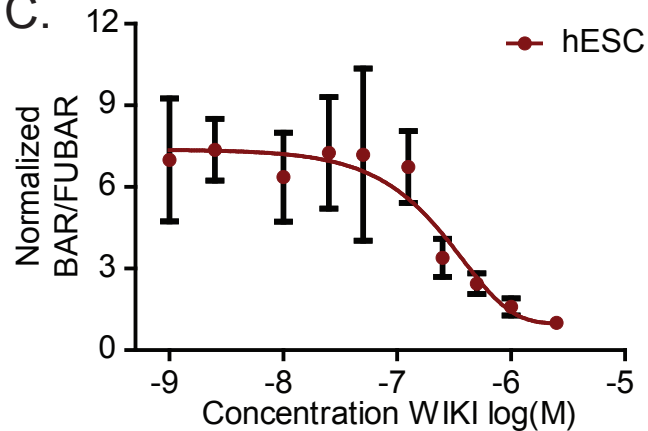

D.

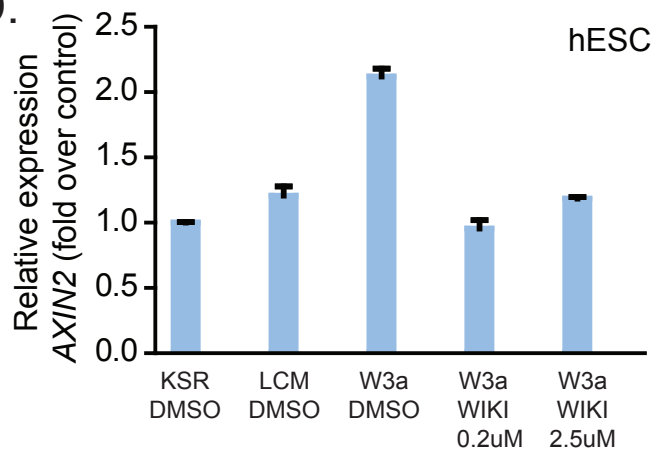

E.

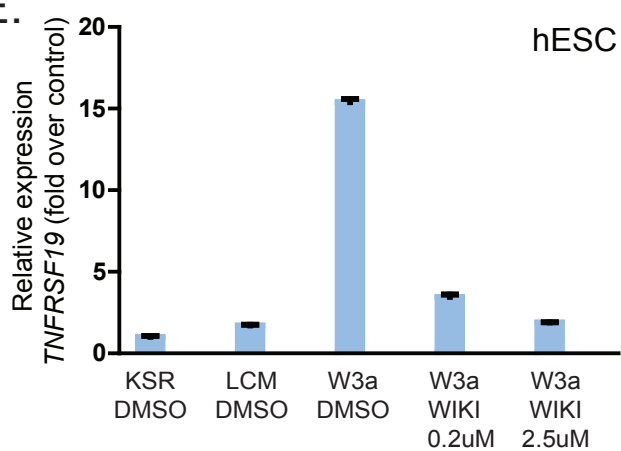

Supplement: Figure S1 — WIKI4 inhibits Wnt/ß-catenin signaling in several cell types. (A) A375 melanoma cells stably expressing the ß-catenin activated reporter were stimulated with an EC20 dose of Wnt3A and treated with the indicated doses of U0126, Riluzole, GSK3 inhibitor IX (left), and XAV-939 (right). The fold change in reporter activity was plotted and the calculated Z-factor (z′) for each compound is indicated above the bars. (B) A heat map showing the inhibitory effects of WIKI4 on Wnt3A-dependent activation of the ß-catenin activated reporter stably transduced in NALM6 B cells, U2OS osteosarcoma cells, and A375 melanoma cells. The results of the firefly luciferase and resazurin activity were normalized to the Wnt3A-stimulated condition, log2 transformed and plotted. (C) H1 hESCs were stably transduced with the ß-catenin activated reporter (BAR) or a mutated ß-catenin activated reporter (FUBAR) driving firefly luciferase. Both cell lines were stimulated with 50% (vol/vol) Wnt3A CM for three days in the presence of a dose curve of WIKI4. The cells were lysed and the ratio of luciferase from the BAR and FUBAR cells was calculated and plotted. (D, E) Wnt3A-dependent increases in the steady-state abundance of gene targets of the Wnt/ß-catenin pathway were prevented by concurrent treatment with WIKI4. hESCS were stimulated for three days with the indicated conditions. The cells were lysed and processed for qPCR for AXIN2 (D) and TNFRSF19 (E). The data was normalized to 100,000 copies of GAPDH and plotted as a ratio to the treatment cultured in KSR media. (PDF) [file pone.0050457.s001.pdf]

**A.** DLD1 colorectal cancer cells

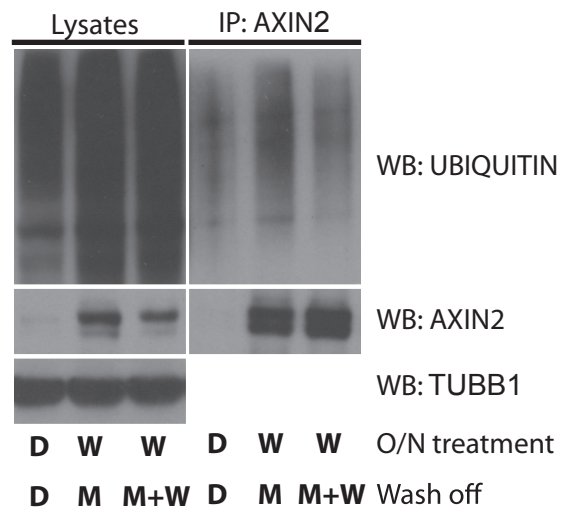

**B.** DLD1 colorectal cancer cells

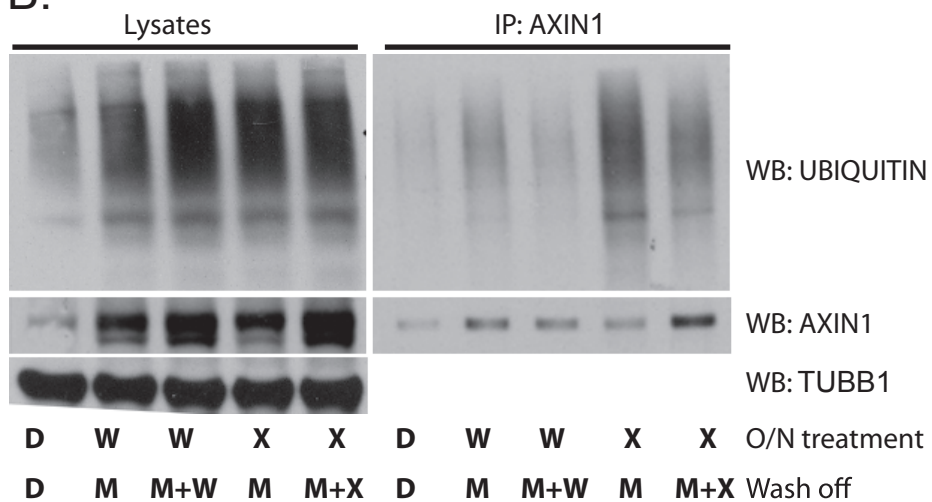

Supplement: Figure S3 — WIKI4 inhibits polyubiquitylation of AXIN proteins in DLD1 colorectal carcinoma cells. WIKI4 inhibits ubiquitylation of AXIN2 (A) and AXIN1 (B) in DLD1 colorectal carcinoma cells. DLD1 cells were treated overnight with DMSO (D), 2.5 µM WIKI4 (W) or 2.5 µM XAV-939 (X). Following a brief wash, the cells were then incubated for two hours with DMSO (D), 10 µM MG132 (M) or MG132 and one of the Wnt/ß-catenin pathway inhibitors. Lysates and AXIN2 (A) or AXIN1 (B) immunoprecipitates from this experiment were processed for western blotting with the indicated antibodies. (PDF) [file pone.0050457.s003.pdf]
