# Supplementary material for: WIKI4, a Novel Inhibitor of Tankyrase and Wnt/ß-Catenin Signaling
Source: PLoS One. 2012 Dec 5;7(12):e50457. doi: 10.1371/journal.pone.0050457 (PMC3515623; doi:10.1371/journal.pone.0050457)
Supplement: Figure S2 — Structure activity relationship of WIKI4 analogs. The Wnt/ß-catenin inhibitory activity of several WIKI4 analogs was tested. The portion of the molecule that is held constant throughout the analysis is depicted in the left panels and the structure specific to each indicated analog is depicted in the right panels. DLD1 colorectal carcinoma cells stably expressing the ß-catenin Activated Reporter (BAR) were treated with a dose escalation of the indicated WIKI analogs. If the compound inhibited signaling, the full dose response curve is depicted, if the compound exhibited no activity, “no response” was indicated, and if the data we have for the compound came from the primary screen, its activity at 330 nM was indicated. (A) Modification of the triazole of WiKI4. (B) Modification of the 1,8-naphthalimide of WIKI4. (PDF) [file pone.0050457.s002.pdf]

Figure S2. Structure activity relationship of WIKI4 analogs.

A.

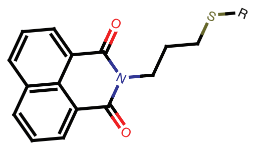

Structure	Fold Wnt reporter	Structure	Fold Wnt reporter
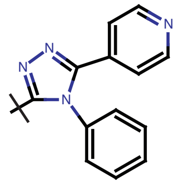 WIKI1	.7 fold at 330nM	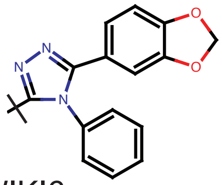 WIKI6	No Response
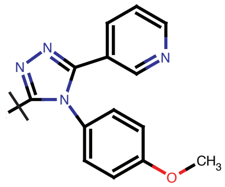 WIKI2	.65 fold at 330nM	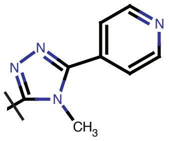 WIKI7	No Response
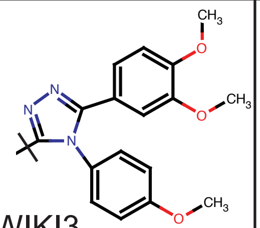 WIKI3	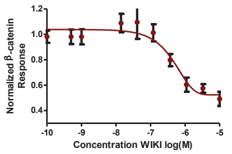	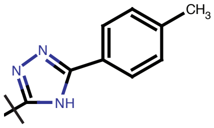 WIKI8	No Response
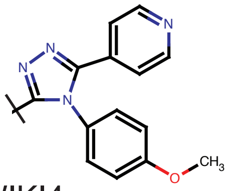 WIKI4	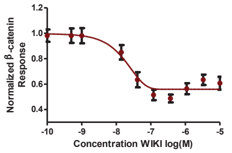	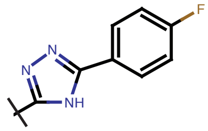 WIKI9	No Response
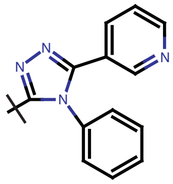 WIKI5	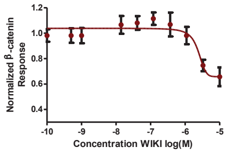	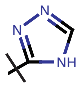 WIKI10	No Response

B.

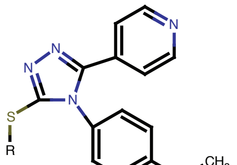

Structure	Fold Wnt reporter	Structure	Fold Wnt reporter
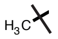 WIKI11	No Response	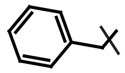 WIKI12	No Response
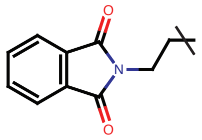 WIKI13	No Response		
