# Supplementary material for: WIKI4, a Novel Inhibitor of Tankyrase and Wnt/ß-Catenin Signaling
Source: PLoS One. 2012 Dec 5;7(12):e50457. doi: 10.1371/journal.pone.0050457 (PMC3515623; doi:10.1371/journal.pone.0050457)
Supplement: Table S1 — Quantitative PCR primers and siRNA sequences. (DOCX) [file pone.0050457.s004.docx]

**Table S1. Quantitative PCR primer and siRNA sequences**.

| **Gene** | **Primer -forward** | **Primer -reverse** |
| --- | --- | --- |
| *AXIN1* | CTGGATACCTGCCGACCTTA | CCGGCATTGACATAATAGGG |
| *AXIN2* | GCGATCCTGTTAATCCTTATCAC | AATTCCATCTACACTGCTGTC |
| *GAPDH* | TGAAGGTCGGAGTCAACGGA | CCATTGATGACAAGCTTCCCG |
| *TNFRSF19* | GGAGTTGTCTAAGGAATGTGG | GCTGAACAATTTGCCTTCTG |
|  |  |  |
| **Gene** | **siRNA -forward** | **siRNA -reverse** |
| *CTNNB1* | GGAUGUUCACAACCGAAUUtt | AAUUCGGUUGUGAACAUCCtt |
| *Ambion-control 1* | - | - |
