# Supplementary material for: WIKI4, a Novel Inhibitor of Tankyrase and Wnt/ß-Catenin Signaling
Source: PLoS One. 2012 Dec 5;7(12):e50457. doi: 10.1371/journal.pone.0050457 (PMC3515623; doi:10.1371/journal.pone.0050457)
Supplement: Table S2 — Compounds tested for structure activity relationship. (DOCX) [file pone.0050457.s005.docx]

**Table S2. Compounds tested for Structure Activity Relationship.**

| **Name** | **UW-ID** | **Company** |
| --- | --- | --- |
| WIKI1 | 1123305 | Chembridge |
| WIKI2 | 1123779 | Chembridge |
| WIKI3 | N/A | Princeton Biomolecular Research |
| WIKI4 | 1123710 | Chembridge |
| WIKI5 | N/A | Princeton Biomolecular Research |
| WIKI6 | N/A | Chembridge |
| WIKI7 | 1123571 | Chembridge |
| WIKI8 | N/A | Princeton Biomolecular Research |
| WIKI9 | 1123676 | Chembridge |
| WIKI10 | N/A | Chembridge |
| WIKI11 | N/A | Chembridge |
| WIKI12 | N/A | Chembridge |
| WIKI13 | N/A | Chembridge |
