# Supplementary material for: WIKI4, a Novel Inhibitor of Tankyrase and Wnt/ß-Catenin Signaling
Source: PLoS One. 2012 Dec 5;7(12):e50457. doi: 10.1371/journal.pone.0050457 (PMC3515623; doi:10.1371/journal.pone.0050457)
Supplement: Table S3 — WIKI analogs identified from public databases. (DOCX) [file pone.0050457.s006.docx]

**Table S3. WIK14 analogs identified from public databases.**

| **Compound: SMILES** | **MW** | **eMolecules ID** |
| --- | --- | --- |
| CCSc1nnc(c2ccncc2)n1c1ccc(OC)cc1 | 312.39 | 3438396 |
| O=c1c2cccc3cccc(c(=O)n1C1CCS(=O)(=O)C1)c23 | 315.34 | 1292932 |
| N#CCSc1nnc(c2ccncc2)n1c1ccc(OC)cc1 | 323.37 | 3438196 |
| CCCSc1nnc(c2ccncc2)n1c1ccc(OC)cc1 | 326.42 | 3441348 |
| OC(=O)CSCCCn1c(=O)c2cccc3cccc(c23)c1=O | 329.37 | 9968189 |
| O=c1c2cccc3cccc(c(=O)n1CCCSc1nnc[nH]1)c23 | 338.38 | 2011452 |
| C=CCSc1nnc(c2ccncc2)n1c1ccc(OCC)cc1 | 338.43 | 1725128 |
| COc1ccc(cc1)n1c(SCC(C)C)nnc1c1ccncc1 | 340.44 | 3523114 |
| COc1ccc(cc1)n1c(SCC(=O)N)nnc1c1ccncc1 | 341.39 | 3525789 |
| OCCSc1nnc(c2ccncc2)n1c1ccc(OCC)cc1 | 342.42 | 13462416 |
| Cn1cnnc1SCCCn1c(=O)c2cccc3cccc(c1=O)c23 | 352.41 | 2011122 |
| CCCCSc1nnc(c2ccncc2)n1c1ccc(OCC)cc1 | 354.47 | 29260864 |
| CCOc1ccc(cc1)n1c(SCC(=O)N)nnc1c1ccncc1 | 355.41 | 1146678 |
| CCOc1ccc(cc1)n1c(SCC(=O)O)nnc1c1ccncc1 | 356.40 | 838257 |
| COc1ccc(cc1)n1c(SCCC(=O)O)nnc1c1ccncc1 | 356.40 | 2011598 |
| COC(=O)CSc1nnc(c2ccncc2)n1c1ccc(OC)cc1 | 356.40 | 2212637 |
| COc1ccc(cc1)n1c(nnc1c1ccncc1)SC1CCCC1=O | 366.44 | 3441354 |
| COc1ccc(cc1)n1c(SCc2ccccc2)nnc1c1ccncc1 | 374.46 | 1392533 |
| COc1ccc(cc1)n1c(nnc1c1ccncc1)SCC(=O)C(C)(C)C | 382.48 | 3420305 |
| COc1ccc(cc1)n1c(SCC2CCOCO2)nnc1c1ccncc1 | 384.45 | 3668677 |
| COc1ccc(cc1)n1c(SCc2ccc(C)cc2)nnc1c1ccncc1 | 388.48 | 1392531 |
| COc1ccc(cc1)n1c(SCCc2ccccc2)nnc1c1ccncc1 | 388.48 | 1994405 |
| COc1ccc(cc1)n1c(SCc2c(C)noc2C)nnc1c1ccncc1 | 393.46 | 3486521 |
| COc1ccc(cc1)n1c(SCc2csc(C)n2)nnc1c1ccncc1 | 395.50 | 3666321 |
| CCOc1ccc(cc1)n1c(SCC2CCCCO2)nnc1c1ccncc1 | 396.51 | 1146758 |
| COc1ccc(cc1)n1c(SCC(=O)c2ccccc2)nnc1c1ccncc1 | 402.47 | 1998313 |
| COc1ccc(cc1)n1c(SCC(=O)NC2CCCC2)nnc1c1ccncc1 | 409.51 | 6615055 |
| CCOc1ccc(cc1)n1c(SCc2ccc(C#N)cc2)nnc1c1ccncc1 | 413.49 | 1146756 |
| COc1ccc(cc1)n1c(SCc2nc3ccccn3c2)nnc1c1ccncc1 | 414.48 | 3440223 |
| O=c1n(CCCSc2nnnn2c2ccccc2)c(=O)c2cccc3cccc1c23 | 415.47 | 2011662 |
| COc1ccc(cc1)n1c(SCc2cccc3ccccc23)nnc1c1ccncc1 | 424.52 | 8232645 |
| COc1ccc(cc1)n1c(nnc1c1ccncc1)SC(C(C)C)C(=O)NC(=O)N | 426.49 | 24957155 |
| O=C1c2ccccc2C(=O)N1CCSc1nnc(c2ccncc2)n1c1ccccc1 | 427.48 | 2387415 |
| Cc1ccc(cc1)c1nnc(SCCCn2c(=O)c3cccc4cccc(c2=O)c34)[nH]1 | 428.51 | 5516592 |
| Cn1c(SCCCn2c(=O)c3cccc4cccc(c2=O)c34)nnc1c1ccncc1 | 429.49 | 2011526 |
| Fc1ccc(cc1)c1nnc(SCCCn2c(=O)c3cccc4cccc(c2=O)c34)[nH]1 | 432.47 | 2011408 |
| Cn1c(SCCCn2c(=O)c3cccc4cccc(c2=O)c34)nnc1c1cccs1 | 434.53 | 2011614 |
| COc1ccc(cc1)n1c(SCC(=O)Nc2c(C)n[nH]c2C)nnc1c1ccncc1 | 435.50 | 3666323 |
| CCOC(=O)CNC(=O)CSc1nnc(c2ccncc2)n1c1ccc(OCC)cc1 | 441.50 | 1146776 |
| O=C1c2ccccc2C(=O)N1CCCSc1nnc(c2ccncc2)n1c1ccccc1 | 441.51 | 2383181 |
| CCn1c(SCCCn2c(=O)c3cccc4cccc(c34)c2=O)nnc1c1cccnc1 | 443.52 | 4988847 |
| Nc1nc(SCCCn2c(=O)c3cccc4cccc(c34)c2=O)nc2sc(C)c(C)c12 | 448.56 | 2404947 |
| Clc1ccc(cc1)c1nnc(SCCCn2c(=O)c3cccc4cccc(c2=O)c34)[nH]1 | 448.92 | 2011256 |
| COc1ccc(cc1)n1c(nnc1c1ccncc1)SC(C)C(=O)NC(C)c1ccccc1 | 459.56 | 29943850 |
| CCOc1ccc(cc1)n1c(SCC(=O)c2ccc3ccccc3c2)nnc1c1ccncc1 | 466.55 | 1146832 |
| COc1ccc(cc1)n1c(SCCCN2C(=O)c3ccccc3C2=O)nnc1c1ccncc1 | 471.53 | 2207542 |
| Cn1c(SCCCn2c(=O)c3cccc4cccc(c2=O)c34)nnc1c1ccc2OCOc2c1 | 472.52 | 5635117 |
| COc1ccc(cc1)n1c(nnc1c1ccncc1)SCC(=O)c1ccccc1Br | 481.36 | 17015308 |
| O=C1c2ccccc2C(=O)N1CCCSc1nnc(c2ccc3OCOc3c2)n1c1ccccc1 | 484.53 | 4402665 |
| CCOc1ccc(cc1)n1c(nnc1c1ccncc1)SCC(=O)N1CC(=O)Nc2ccccc12 | 486.55 | 3505641 |
| COc1cc(ccc1OC)c1nnc(SCCCn2c(=O)c3cccc4cccc(c2=O)c34)n1C | 488.56 | 2011794 |
| O=c1c2cccc3cccc(c(=O)n1CCCSc1nnc(c4ccncc4)n1c1ccccc1)c23 | 491.56 | 2011064 |
| O=c1n(CCCSc2nnc(c3cccnc3)n2c2ccccc2)c(=O)c2cccc3cccc1c23 | 491.56 | 5516594 |
| O=c1n(CCCSc2nnc(c3cccs3)n2c2ccccc2)c(=O)c2cccc3cccc1c23 | 496.60 | 4955721 |
| CCOc1ccc(cc1)n1c(SCC(=O)Nc2cc(C)c(Cl)cc2OC)nnc1c1ccncc1 | 510.01 | 1146810 |
| COc1ccc(cc1)n1c(SCCCn2c(=O)c3cccc4cccc(c2=O)c34)nnc1c1cccnc1 | 521.59 | 2011658 |
| COc1ccc(cc1)n1c(SCCCn2c(=O)c3cccc4cccc(c2=O)c34)nnc1c1ccncc1 | 521.59 | 2011774 |
| O=c1n(CCCSc2nnc(C3COc4ccccc4O3)n2c2ccccc2)c(=O)c2cccc3cccc1c23 | 548.61 | 4988849 |
| COc1cc(ccc1OC)c1nnc(SCCCn2c(=O)c3cccc4cccc(c2=O)c34)n1c1ccccc 1 | 550.63 | 5518152 |
| COc1ccc(cc1)n1c(SCCCn2c(=O)c3cccc4cccc(c2=O)c34)nnc1c1ccc(OC)c(OC)c1 | 580.65 | 5518138 |
| COc1ccc(cc1)n1c(SCCCn2c(=O)c3cccc4cccc(c2=O)c34)nnc1COc1ccc2OCOc2c1 | 594.64 | 5634633 |
| COc1ccc(cc1)n1c(SCCCn2c(=O)c3cccc4cccc(c2=O)c34)nnc1c1sc2ccccc2c1Cl | 611.13 | 5516600 |
